# The scent of infanticide risk? Behavioural allocation to current and future reproduction in response to mating opportunity and familiarity with intruder

**DOI:** 10.1007/s00265-018-2585-4

**Published:** 2018-10-18

**Authors:** J. A. Eccard, D. Reil, R. Folkertsma, A. Schirmer

**Affiliations:** 1 Animal Ecology, Institute of Biochemistry and Biology, University of Potsdam, Potsdam, Germany; 2 Present Address: Vertebrate Research, Institute for Plant Protection in Horticulture and Forests, Julius Kühn-Institute, Münster, Germany; 3 Evolutionary Adaptive Genomics, Institute for Biochemistry and Biology, University of Potsdam, Potsdam, Germany

**Keywords:** Reproductive strategy, Infanticide risk, Odour recognition, Familiarity, Counterstrategy, Sexual conflict

## Abstract

**Abstract:**

The killing of young by unrelated males is widespread in the animal kingdom. In short-lived small rodents, females can mate immediately after delivery (post-partum oestrus) and invest in future reproduction, but infanticide may put the nestlings, their current reproductive investment, at risk. Here, we investigated the behavioural trade-offs between mating interest and nest protection in an arena experiment with bank voles (*Myodes glareolus*). Non-gravid females (*n* = 33) were housed at one end of a large structured arena with their nestlings. Different scents (cage bedding) were presented to each female in a replicated design. Three combinations of mating opportunities and male-female familiarity were simulated using different scent donors: mating opportunity with the sire of the nestlings with whom the female was familiar; mating opportunity with a male unrelated to the offspring and unfamiliar to the female, thus posing a higher risk to the offspring; and neither risk nor mating opportunity (clean control). Most females investigated male scents, regardless of familiarity, leaving their litter unprotected. During control treatment, females with larger litters spent less time at the scent area, indicating increasing nursing demands or better protection. Females with older litters visited scents more often, suggesting an increased interest in reproduction while they are non-gravid alongside the decreased risk of infanticide for older young. In the presence of unfamiliar scents, females spent more time protecting their nests, supporting the perceived association of unfamiliarity with infanticide risk. Thus, rodent females flexibly allocate time spent between searching for a mate and protecting their nest, which is modulated by their familiarity with a potential intruder.

**Significance statement:**

Infanticide by conspecific males is an extreme form of sexual conflict and has large costs on females, abolishing their investment into current offspring. In an experimental approach, we exposed lactating female bank voles to different combinations of mating opportunity and familiarity to a (simulated) intruder: (1) the sire of the nestlings with whom the female was familiar and, therefore, potentially less risky in terms of infanticide; (2) a male which was unrelated and unfamiliar to the female and thus posed a higher risk to the offspring; or (3) as a control, cage bedding, which posed neither risk of infanticide nor a mating opportunity. We show that females flexibly allocated pup protection and mating interest based on their familiarity with the male, indicating that the unfamiliar males pose a threat to offspring, which is perceived by the females. Females further adjusted their behaviour to the size and/or age of their current litter, investing more time in male scents when offspring were older, thus balancing future and current investments into reproduction.

**Electronic supplementary material:**

The online version of this article (10.1007/s00265-018-2585-4) contains supplementary material, which is available to authorized users.

## Introduction

Infanticide, the killing of conspecific young, is a widespread behavioural trait across mammals. Infanticide by males is an extreme form of sexual conflict. While the male may increase its share of offspring in the next generation, the female loses a large reproductive investment. Therefore, it should be beneficial for females to minimise or avoid infanticide. Infanticide is common in species with harem structures and strong reproductive skew among males (Lukas and Huchard 2014), giving the perpetrator access to reproduction with the female that lost its offspring (Hrdy 1979) and limiting the reproductive success of male competitors that sired the killed offspring (Agrell et al. 1998; Ebensperger 1998; Ebensperger and Blumstein 2007; Hrdy 1979). In monogamous and polygynandrous species, the adaptive value of this male trait is less clear. In many polygynandrous rodent species, females mate directly after delivering young (post-partum oestrus), slightly delay implantation, and simultaneously invest energy into both lactation and the new pregnancy. The loss of young advances the date of birth of the new litter (Gustafsson et al. 1980), and the size of the new litter can be larger (Elwood and Kennedy 1990).

Several counterstrategies among females against male infanticide have been identified (summarised in Lukas and Huchard 2014), including promiscuity to confuse paternity, directly attacking potential perpetrators (Ylönen and Horne 2002), avoiding infanticidal individuals, aggression, and territoriality (Elwood and Kennedy 1990; Palanza et al. 1994; Agrell et al. 1998), as well as early termination of pregnancy to reduce the potential damage (Bruce effect, Bruce 1959; Eccard et al. 2017). Counterstrategies against infanticide come with a cost for the female, however, as these increase the risk of injury and energetic investments (Agrell et al. 1998; Ylönen and Horne 2002). Therefore, the effort allocated to counterstrategies should be adjusted to the actual risk that a conspecific poses, and individuals would benefit from estimating risk of infanticide and adjusting their behaviour accordingly. For example, female mice were observed to predominantly attack infanticidal males compared to non-infanticidal males and are more likely to abort pregnancy if exposed to infanticidal males (Elwood and Kennedy 1990, Elwood et al. 1990). Meanwhile, bank vole females attack all unfamiliar conspecifics and successfully defend their offspring, since 30% of conspecific voles exhibit infanticidal behaviour (Ylönen and Horne 2002).

The cost of reproduction is a common life history trade-off, with an increased energetic investment into the current reproduction coming at the expense of future (Stearns 1989). For iteroparous species, the reproductive trade-off between current and future reproduction is especially pronounced when already caring for offspring (Koivula et al. 2003). This is the case for many rodent species, where females can become pregnant while already caring for an existing litter, and thus trade-off nursing and protecting their young against mating opportunities (Klemme et al. 2011). Previous studies revealed that this trade-off might be modulated by the size of investment into the current reproduction, e.g. increasing with litter size (Jonsson et al. 2002a) or, conversely, by vulnerability to infanticide, with younger offspring needing more protection (Koskela et al. 2000). Further, with rodents being short-lived iteroparous mammals experiencing high predation pressure and low chances to survive until the future offspring are weaned, the current offspring may be of higher value than potential future offspring. Nevertheless, mate search is of similar importance as nest guarding and, therefore, might affect behavioural activities outside the nest.

In this study, we aim to explore the flexibility and constraints of reproductive behaviour in response to risk of infanticide by unfamiliar males on the one hand, and mating opportunities on the other. As a novelty, we studied time allocation of females and measured both nest protection behaviour and investigation of male scent in the same arena by providing space and time on larger scales than many earlier experiments. By using large, structured arenas, we forced the females to move out of reach and sight from their nest in order to investigate the male scent, allowing us to separate nest-guarding behaviour from investigation of potential mates, and to study the female’s time allocation to either area. Under the assumption that non-gravid females of any short-lived rodent species with high predation risk and high reproductive rates should be interested in mating and reproduction, we created a trade-off between future reproduction (mating interest) and current reproduction (litter threatened by infanticide risk from an unfamiliar male) by mimicking the presence of a mating partner with a scent treatment. It is generally assumed that male rodents visit females and that females do not actively search for males. Meanwhile, we know from our previous studies that vole females actively visited mating partners that were confined in compartments (Klemme et al. 2011), actively follow artificial scent trails of males in outdoor experiments (Breedveld et al., unpublished data), and that tree rat males and females met at mating places far away from female’s nests (Eccard et al. 2004; Eccard et al., unpublished data). We thus assume that rodent females take an active part in mate search.

Here, we exposed female non-gravid bank voles (*Myodes glareolus*) with varying current reproductive investment to three different treatments which differed in male to female familiarity and mating opportunity. Assuming that females’ familiarity to the scent donor might affect perceived infanticide risk, we hypothesise that we should find differences in nest-guarding behaviour, e.g. presence at the nest. To test whether females actually experience allocation conflicts between offspring protection and mating interest when presented with a male, we also used a control that allows us to monitor female time allocation at the nest and the scent area without a potential mating partner. By providing soiled cage bedding from different scent donors and a clean control, three combinations of familiarity and mating opportunities were simulated. Mating opportunity with high familiarity and lower infanticide risk was simulated using the sire of the nestlings as scent donor (sire treatment), mating opportunity with no familiarity and higher infanticide risk by using a male unrelated to the offspring and unfamiliar to the female (unknown treatment), and neither risk nor mating opportunity by using a clean cage bedding (control treatment). If females use familiarity as a cue for infanticide risk, we would expect there to be differences in behaviour of females in the unknown treatment compared to the other two treatments. If mating interest alone drives the behaviour of the females, however, we would expect behaviour in the two male scent treatments to differ from the control treatment. As the importance of the two interests may vary with the investment into the current litter, we also tested the effects of litter size and litter age on treatment effects.

In the study species, infanticidal behaviour seems to be common. Both sexes show infanticidal behaviour and about 30% of individuals in a population were measured to be infanticidal (Ylönen et al. 1997; Ylönen and Horne 2002). Infanticide has a large impact on breeding success, juvenile recruitment, and population development (Ylönen et al. 1997; Poikonen et al. 2008; Korpela et al. 2010; Opperbeck et al. 2012). Although male bank voles are unable to identify their own offspring from unrelated ones, familiarity, copulation, and temporary association with the mother inhibits infanticide by the males (Vihervaara et al. 2010, similar findings in wild house mice: McCarthy and Vom Saal 1986). Female bank voles exhibit common counterstrategies to infanticide, such as nest defence and promiscuity to obscure the paternity of their offspring (Koskela et al. 1997; Mappes et al. 1995; Klemme and Ylönen 2009; Klemme et al. 2011). Females may not differentiate between the infanticidal status of a male, but are able to distinguish their former mate from an unfamiliar male (Kruczek 1998), which Kruczek interprets as a response to reduced infanticide risk. Additionally, in a lab study, a third of bank voles exhibited infanticidal behaviour against the unprotected nests of unfamiliar females, and if the females were present at the nest site all, unfamiliar intruders were met with high levels of aggression and no pups were killed (Ylönen and Horne 2002). Thus, female bank voles may use familiarity as a cue for infanticide risk.

Female bank voles are polyestrous with one oestrus cycle lasting 4 days, including a mating period (cycling oestrus CE or behavioural oestrus, Klemme et al. 2011). Cycling oestrus can also be triggered by male odour (as in *Microtus* voles: Carter et al. 1980; Newman and Halpin 1988) or the presence of males in the same room. Inexperienced females, which were in the proximity of a male, had higher fertility compared to females that were kept singly (Westlin and Gustafsson 1983). After successful pregnancy, a post-partum oestrus (PPE) occurs, when females are receptive in a short time period after parturition, followed by concurrent lactation and gestation (Klemme et al. 2011). In bank voles, as in many rodents, ovulation is triggered by mating (induced ovulation). During PPE, a single, brief mating is sufficient for conception, while females engage in extensive courting and mating activity during CE, subsequently mating with all available males (Klemme et al. 2011). PPE usually results in females being gravid while lactating, which increases the average gestation period from 19–21 days (after CE) to 22–24 days (Brambell and Rowlands 1936; Gustafsson et al. 1980).

There is little and equivocal information available on whether female bank voles can conceive during lactation. Gustafsson et al. (1980, 1983) assumed lactational anoestrus in a laboratory colony of caged bank vole pairs, since only 2% of females (*n* = 640) conceived during lactation. Meanwhile, in semi-wild experimental bank vole populations in large outdoor enclosures, we observed that 40% of non-gravid, lactating females (Eccard and Ylönen 2003, *n* = 39 females) and 9 out of 10 of non-gravid, lactating females (re-analysing data reported in Eccard et al. 2017) were conceiving within days after being release to enclosures, while still accompanied by their several days-old litter.

Although sample sizes vary greatly and further research is needed, we assume that female bank voles are not anoestric during lactation, but in a cycling oestrus. In cycling oestrus, females have to mate repeatedly (Klemme et al. 2011) and, if possible, with multiple males to induce ovulation (e.g. Klemme et al. 2011). Mating and courtship behaviour in voles includes a lot of time and chasing (Klemme et al. 2011). In a caged situation, the courting pair would be moving through the nest, disturbing the females’ offspring and triggering aggressive behaviour in the female in defense of her pups (Koskela et al. 1997). This conflict offers an alternative explanation for low breeding success among nursing females observed in caged pairs, while in nature, females can mate outside the nest. Post-partum oestrus females, in contrast, need to mate only briefly and only once (Klemme et al. 2011) to conceive, making successful matings in a small cage possible. Based on the information above, we assume that non-gravid, lactating females are interested in information on mating opportunities.

## Material and methods

The experiment was conducted using 33 adult female bank voles and their litters in the summer and autumn of 2013 and 2014. Wild bank voles were captured at three locations around Potsdam, Germany. Females were housed individually in standard Makrolon cages (Ehret GmbH Germany, type III: 42 cm × 27 cm × 16 cm), containing saw dust and hay as bedding, as well as a wooden nest box and a paper rolls for shelter. Water and food pellets (ssniff® NM, ssniff® R/M-H Ered II) were available ad libitum. Animals were marked individually via passive integrated transponders (Trovan AEG). Non-gravid females were paired with a male for 10 days to obtain litters with known sires. We used this longer period to ensure that females were familiar with the sire of the litter. After separation, sires were kept in individual cages to provide soiled cage beddings to use as scent in the experiment.

The pregnancy status of females was determined via visual examination and weight development. Approximately 18–20 days after pairing, females gave birth to litters with a size of 1–8 offspring with an average litter size of 4 ± 1 (mean ± standard deviation (SD)). At an offspring age of 1–12 days, the nest box containing the female and her litter was transferred to the experimental arena. We distributed offspring ages and litter sizes evenly among the three scent treatments. Females and their litters were habituated to the arena for 1 day. Thus, during treatment, all females were outside post-partum oestrus, which in general implies a reduced mating interest, but this is discussed further in our discussion. Both animal housing and the experimental room were kept at 17–20 °C and a 13:11 L:D light cycle, starting at 7 am. Food pellets and water were provided ad libitum behind the first intermediate wall from the nest site.

**Fig. 1:**
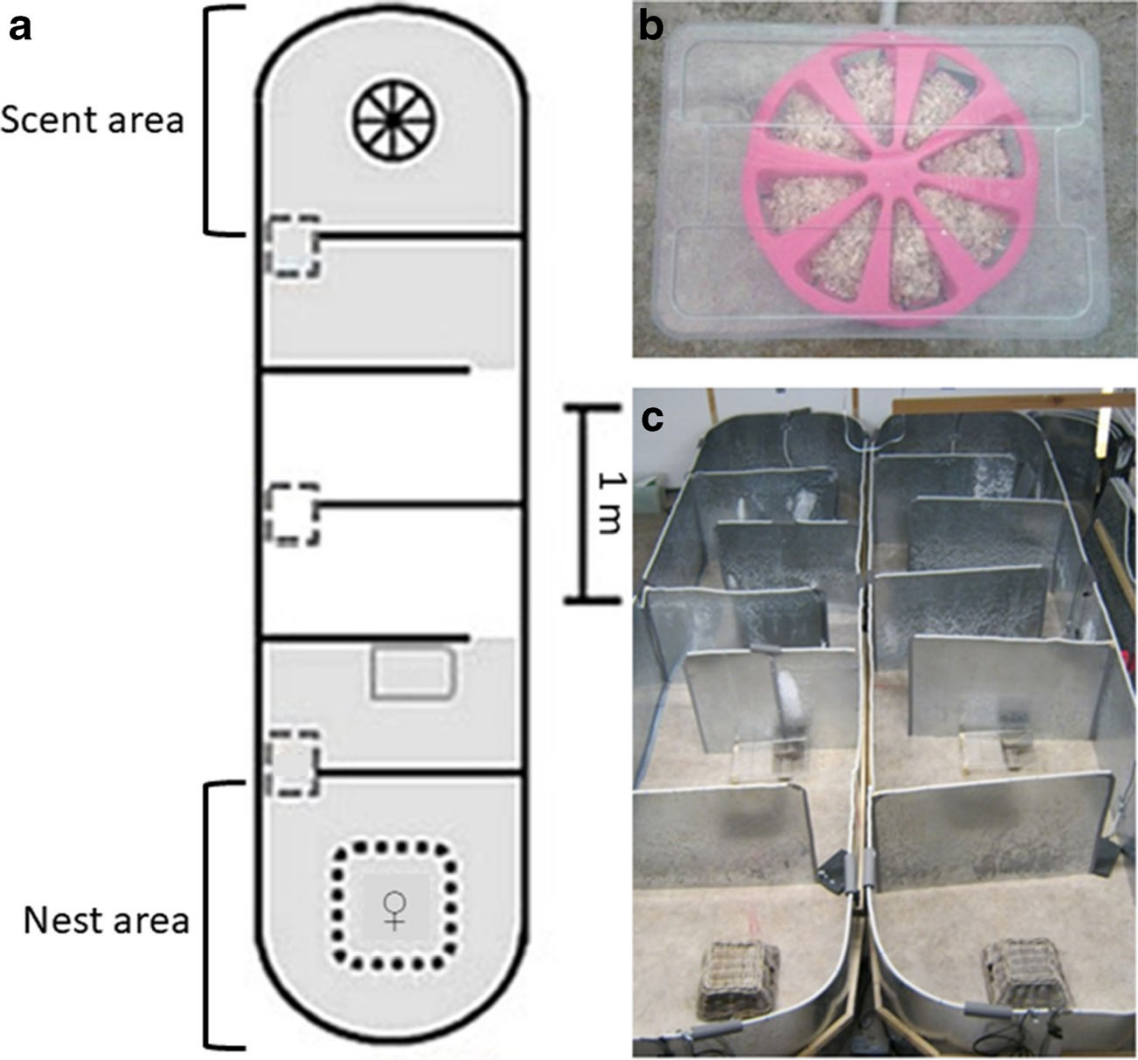
Experimental set-up. **a** Schematic plan of one arena, the grey areas show the visual fields of the cameras; scent wheel (black circle) and wooden nest box (dotted square) sheltering the female and her litter were placed at opposite ends; food and water supply (grey rectangle) was located behind the first intermediate wall. Plate antennas were located under the nest box and the scent wheel and at the entrance to the nest area and scent area respectively as well as in the middle of the arena (dashed rectangles). **b** Scent wheel under plastic lid. **c** Set-up of two neighbouring metal arenas

The experimental set-up consisted of two indoor arenas positioned side by side, measuring 5.0 m × 1.3 m × 1.0 m (L × W × H) and containing a nest area and a scent area on opposite ends (Fig. 1). Five intermediate walls measuring 1.1 m × 1.0 m (D × H) were placed at intervals of 0.8 m extending from alternating sites of the arena (Fig. 1) to increase travel distance (slalom) from one end to another and to obstruct the view of the nest. The nest box in the nest area was additionally covered with a wooden basket to create a safe area close to, but outside of the nest box. Nest attendance was monitored with a RFID plate antenna (EuroID, Netherlands) placed under the nest box and, additionally, by camera observation capturing the area outside of and around the nest.

Each female was exposed to cage bedding provided in a scent wheel at the far end of the arena (scent area). Cage bedding came from and contained the odour of the sire of the litter (sire treatment), an unfamiliar male (unknown treatment), or clean cage bedding (control treatment). We collected cage bedding (100 g) from heavily soiled areas (urinary latrines) directly before placing it in the arena. The scent wheel was a circular silicone box (27 cm in diameter, 5 cm in height) with eight separate compartments which were only accessible from outside the wheel via an entrance hole. The presence at the scent wheel and in the scent area was monitored using an RFID plate antenna placed underneath the scent wheel and using camera observations capturing the total scent area including scent wheel. All compartments were filled with a proportion of cage bedding of the same origin. Intensity of investigation by the females could thus be quantified by counting the number of entered compartments.

Two females and their litters were always observed simultaneously in two identical setups. To avoid a mixture of odour cues, we presented the scent of the same male in both arenas, serving as the sire treatment in one arena and as the unknown treatment in the other arena. The next day, the scent of the other litters’ sire was presented ensuring a change in the order of scent treatment per female. Fresh cage bedding serving as a control was presented either the day before or after both male scents, thus resulting in four different treatment orders. Females were equally divided among these four orders.

Prior to treatment females and their litter were placed into the arena and allowed 24-h habituation. Treatments followed on three consecutive days and at the start of each treatment, cage bedding was placed in the scent wheel half an hour prior to the lights turning off, as bank voles have their peak activity phase during twilight (Baumler 1975; Greenwood 1978; Galsworthy et al. 2005). We observed females during a 6-h observation period. Scent wheel and scent area were cleaned between each treatment using a 10% ethanol solution, and arenas were cleaned before each replicate.

### Variables measured at the scent wheel

Using video observations and data from the RFID plate antenna, we measured the latency to visit the scent area for the first time, the time spent in the scent area, the number of visits to the scent area, and the length of each visit to the scent area, and counted the number of entries to compartments of the scent wheel (including re-entries of already visited compartments). The first visit after the start of each treatment was analysed separately from later visits since scent may be volatile and only relevant for the female at first inspection. Furthermore, females may adjust their behaviour after they have gathered additional information by investigating the scent.

### Variables measured at the nest

We used video observations from above the nest area and data from an RFID plate antenna under the nest to measure the latency to leave the nest for the first time after the start of the treatment. We also counted the number of absences from the nest and measured each length of absence. From these measurements, we calculated the mean length of absence, cumulative time absent, and longest absence.

It was not possible to conduct a “blind” analysis of videos, because both data collection and video analysis were conducted by the same person (AS). The presence and absence of RFID tags in females at nest and scent wheel, however, were logged by a machine, blind to treatments.

### Statistical analyses

For each measured variable, a generalised linear mixed model (GLMM) or a linear mixed model (LMM) was constructed. Females were observed repeatedly under each scent treatment in four different treatment orders. Litter size and age of offspring at the start of the experiments were treated as individual constants in the statistical model, since the offspring age of individual females among trials varied only little (3 days) compared to the variation in offspring age among females (12 days). We therefore used mixed models with female ID and treatment order as random factors to account for variation caused by these factors. The contribution of treatment order to the explained variance was negligible for most models, but was kept for the sake of completeness in all models. Male ID was included as a random factor in earlier stages of the analysis, since the same male provided odour for both females on the same day. However, inclusion of male ID did not improve models and was therefore excluded from the analysis presented here. This finding confirms an assumption of the experimental set-up: we aimed to capture perceived infanticide risk based on the female’s familiarity with the male and not based on potential differences in infanticidal tendencies between males.

Scent treatment, offspring age, litter size, and interactions with scent treatment were used as fixed factors. Since many of the variables did not follow a normal distribution, we used GLMMs, modelling the appropriate error structure of the data via the underlying distribution family and corresponding link function. All models were created with either the function *lmer* or *glmer* from the R package ‘lme4’ (Bates 2010). Non-significant two-way interactions were removed from the model if they did not increase model fit (Akaike Information Criterion (AIC) values, Zuur et al. 2009). The most parsimonious model was then used to assess the proportion of explained variance by fixed factors alone (marginal *R*^2^) and fixed and random factors together (conditional *R*^2^) according to Nakagawa and Schielzeth (2013). These values can be seen as goodness-of-fit measures of GLMMs similar to the *R*^2^ value of generalised linear models (Johnson 2014; Nakagawa and Schielzeth 2013), and differences among these *R*^2^ values indicate whether or not the inclusion of the random factor includes the model. The level of significance was set to α < 5%.

#### Data availability

The datasets generated and analysed during the current study are available from the corresponding author on reasonable request.

## Results

In 99 trials observed, females left the nest during the 6-h observation periods in all but two trials. Nest absence was structured into separate bouts. In 72 trials, the females visited the scent area. Descriptive statistics across all trials are given in Supplemental Table S1.

### Variables measured at the nest

**Fig. 2:**
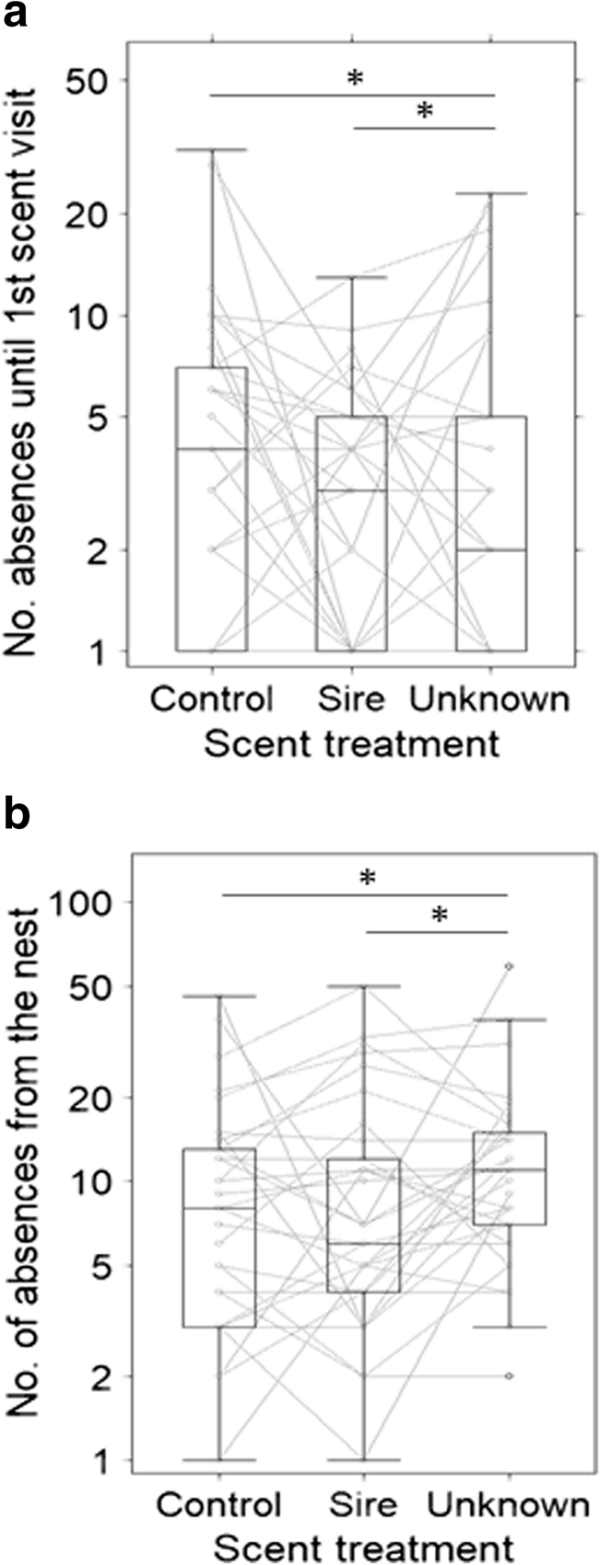
Nest presence (**a** number of absences until females’ first visit to the scent area; **b** total number of absences from the nest during the 6-h observation period) of bank vole females in response to male scent treatment and age of offspring by 33 lactating but not gravid female bank voles (*n* = 99 trials, statistical models in Table 1). Boxes represent the 50% range of the data, the black bar the median and the whiskers the quartiles; stars refer to *p* < 0.05. Grey lines represent the raw measurements of females’ individual responses under each scent treatment

Nest absence differed between the familiar (sire) and unfamiliar (unknown) male, but the sire treatment was not different from the control (Table 1, Figs. 2b and S2). During the first phase of the experiment—before the female had first visited the scent area—the average number ± SD of absences from the nest was lower in the unknown male treatment (3.6 ± 3.0 absences) than in both control (5.7 ± 6.9) and sire treatment (4.8 ± 6.2) (Table 1, Figs. 2a and S1). The opposite was observed when considering the total experimental 6-h observational period: the average number of absences was higher in the unknown male treatment (13.1 ± 11 absences) compared to both control (10.6 ± 10.4) and sire treatment (10.8 ± 11.3) (Table 1, Figs. 2b and S1). Independent of scent treatment, the number of absences before females visited the scent wheel tended to increase with offspring age, while the length of each single absence decreased with offspring age (Table S2).

**Table 1:** Effects of scent treatment and reproductive effort in mixed models of behavioural response variables related to nest presence of 33 vole females in 3 scent treatments (*n* = 99 trials). Given are the model types (lmm: linear mixed model, data transformation (log or boxcox); glmm: generalised lmm, model family (link function)). Rm: marginal *R*^2^ and Rc: conditional *R*^2^. Non-significant two-way interactions were removed from the model (nim: not included in model). Variables that did not allow the model to converge could not be presented (not converged). *P* values indicated are < 0.1(.), < 0.05*, < 0.01**, < 0.001***

Response variable	Number of absences before 1st visit to scent			Number of absences (total) from nest			Latency to leave nest		
Model(family(link))	glmm(poisson(log))			glmm(poisson(log))			lmm(^0.3)		
Rm(%)	7			11			3		
Rc(%)	72			82			43		
Fixed factor	Effect size	Error	*p*	Effect size	Error	*p*	Effect size	Error	*p*
Sire vs control	− 0.17	0.11		0.02	0.07		0.10	0.25	
Unknown vs control	− 0.44	0.12	***	0.21	0.07	**	− 0.08	0.24	
Unknown vs sire	− 0.27	0.12	*	0.18	0.07	**	− 0.19	0.25	
Offspring age	0.05	0.04		0.07	0.04	(.)	− 0.04	0.05	
Litter size	0.01	0.01		Not converged			0.08	0.11	
Figure		2a			2b

Length of single absences (*n* = 1054 absences) and the cumulative duration of absence in the first phase or over the total observation period did not differ between scent treatments or levels of reproductive investment. Furthermore, these models had low explanatory power and are therefore reported in the supplement (Table S2).

### Variables measured at scent area

Most of the behavioural responses measured at the scent area differed between control and both male scent treatments, but there were few differences between the sire and unknown treatment (Tables 2 and S3). Females’ visits at the scent area tended to be more likely if male scent was presented (28 females with unknown male scent, 30 females with sire scent) than in control trials (24 females, Table 2). The latency to visit the scent area tended to be longer in the control treatment compared to both treatments with male scent (Table 2).

**Table 2:** Effects of scent treatment and reproductive effort in mixed models of behavioural response variables related to scent investigation of 33 vole females in three scent treatments. Given are the model types (lmm: linear mixed model, data transformation (log or boxcox); glmm: generalised lmm, model family (link function)), Rm: marginal *R*^2^ and Rc: conditional *R*^2^. Non-significant two-way interactions were removed from the model (nim: not included in model). Variables that did not allow the model to converge could not be presented (not converged). *P* values < 0.1(.), < 0.05*, < 0.01**, < 0.001***

Response variable	Probability to visit the scent			Compartments total			Number of visits to scent			Longest visits at the scent area			Latency to 1st scent visit			Compartments at 1st scent visit		
Model	glmm			glmm			lmm			lmm			lmm			glmm		
Family(link function)	Binomial(probit)			Poisson(log)			(log)			(log)			(^0.5)			Poisson(log)		
Rm(%)	14			26			18			19			5			51		
Rc(%)	38			83			71			30			34			76		
Fixed factor	Effect size	Error	*p*	Effect size	Error	*p*	Effect size	Error	*p*	Effect size	Error	*p*	Effect size	Error	*p*	Effect size	Error	*p*
Sire vs control	1.6	0.8	(.)	1.11	0.12	***	0.32	0.13	*	− 1.09	1.0	**	− 1.52	0.75	(.)	1.99	0.23	***
Unknown vs control	0.9	0.7		1.04	0.12	***	0.55	0.12	***	− 2.06	1.0	**	− 1.53	0.75	(.)	1.61	0.23	***
Unknown vs sire	− 0.7	0.8		− 0.07	0.08		0.24	0.11	*	0.96	1.0		− 0.01	0.75		−0.38	0.23	**
Offspring age	nim			0.07	0.05		0.12	0.04	*	0.06	0.05		− 0.14	0.14		0.01	0.04	
Litter size	nim			Not converged			− 0.01	0.10		− 0.39	0.17		− 0.12	0.35		0.01	0.10	
Sire*litter size (vs control)	nim			nim			nim			0.57	0.24	*	nim			nim		
Unknown*litter size (vs control)	nim			nim			nim			0.68	0.24	*	nim			nim		
Unknown*litter size (vs sire)	nim			nim			nim			0.11	0.24		nim			nim		
Figure				3c			3a, 3b			3d								

**Fig. 3:**
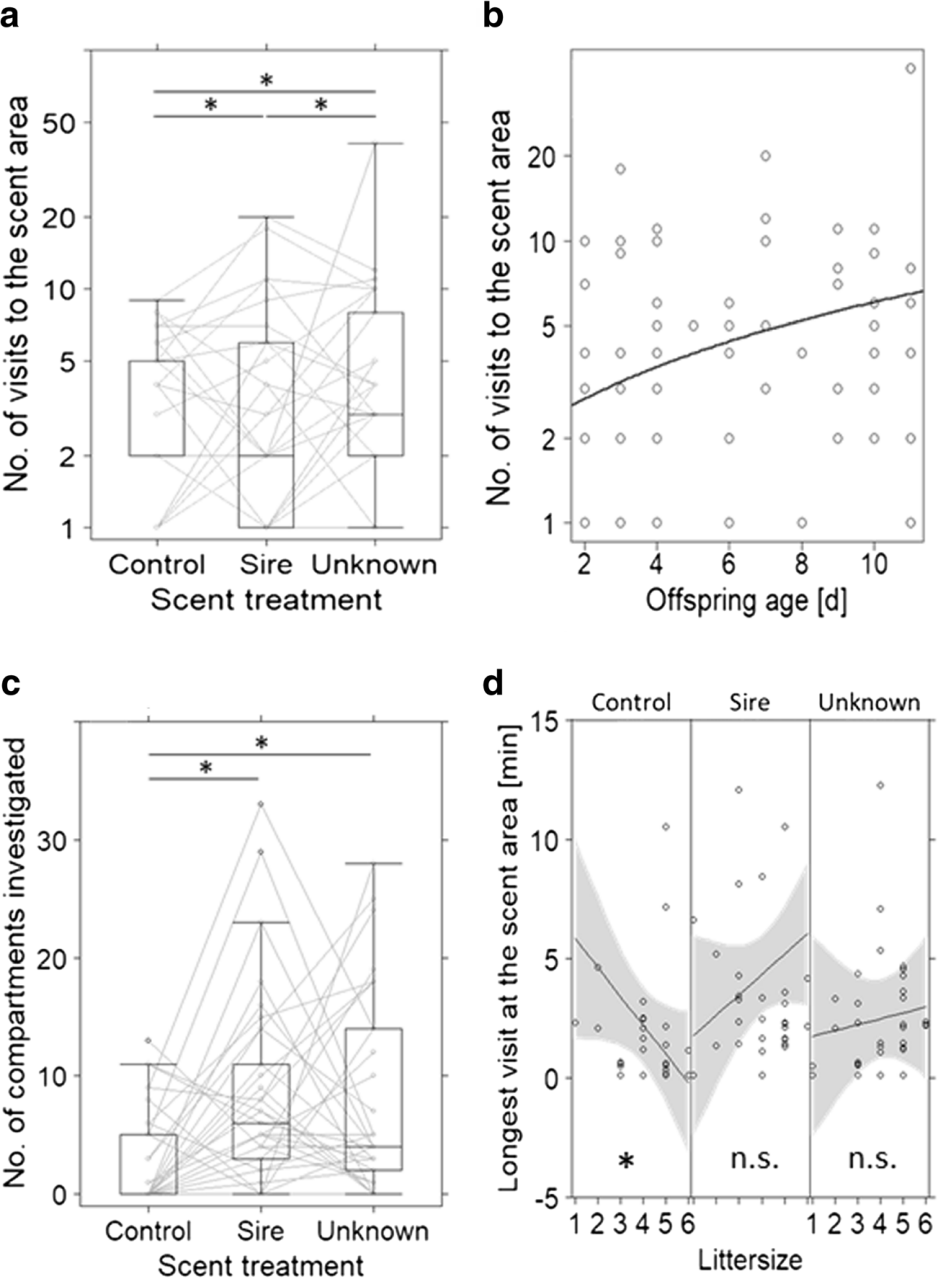
Visitation of scent area (**a** total number of visits to the scent area for each scent treatment; **b** total number of visits to the scent area as a function of offspring age; **c** total number of compartments investigated; **d** longest visit to the scent area for each scent treatment and as a function of offspring age) by 32 lactating but not gravid bank vole females in three different scent treatments (99 trials). Stars refer to post hoc tests with *p* < 0.05 (statistical models in Table 2). Boxes represent the 50% range of the data, the black bar the median and the whiskers the quartiles; grey lines represent the raw measurements of females’ individual responses under each scent treatment. ‘Scent area’ refers to the far end of the arena where the scent wheel is centrally located; compartments refer to the subunits of the scent wheel

The number of compartments in the scent wheel investigated by females differed between the control treatment (2.7 ± 4.0 compartments, Figs. 3c and S1) and sire treatment (8.4 ± 8.2 compartments), as well as the unknown treatment (7.8 ± 8.3 compartments), but responses to the two scents of male treatments did not differ. The total number of visits to the scent area differed among the three treatments, with the scent of an unknown male receiving the most visits and the control receiving the least (Figs. 3a and S1). Across the three treatments, females with older offspring visited the scent area more often than females with younger offspring (Fig. 3b). The longest visit (Fig. 3d) and the total time at the scent area were both best explained by an interaction of scent treatment with litter size (i.e. differences among slopes, Table 2). In the control treatment, females with larger litters spent less time investigating the scent area than females with smaller litters (longest time at scent and total time at scent: post hoc Pearson’s rho > − 0.36, *p* < 0.037, *n* = 33), while in both male scent treatments, a correlation with litter size was not detected (all rho < 0.17, *p* > 0.3; Fig. 3d).

## Discussion

### Nest protection

This study investigated the behaviour of female rodents in response to their familiarity to males, mating opportunity, and varying levels of current reproductive investments. By combining temporal allocation to specific behaviours with spatial allocation at relevant locations, we show that lactating female bank voles increase their presence at the nest site when presented with the scent of unfamiliar males (Fig. 2, Table 1). Nest presence and absence may be a measure of the female’s nest protection, and thus an estimation of infanticide risk perceived by the female. Females stayed longer in the nest when first perceiving unfamiliar male scent from afar (Fig. 2a), and after investigating the scent, they stayed near but outside of the nest for longer compared to the sire or control treatment (Fig. 2b). It thus seems that females are able to recognise the sire of their litter, which is in accordance with findings from Kruczek (1998), and recognise that an unfamiliar male poses a higher infanticide risk compared to the sire male. This supports earlier observations of attacks on unfamiliar conspecifics (Ylönen and Horne 2002) and reduced infanticide risk by stud males (Vihervaara et al. 2010). Besides leaving the nest in order to find food or a mating partner, females may be more successful protecting their litter from outside of the nest since they can attack an intruder before it has located the nest. Rodent females are known to aggressively attack intruders (Agrell et al. 1998; Ylönen and Horne 2002) while having to find a mating partner at the same time. As long as females had not investigated the scent area in detail, they presumably protected their offspring with their presence inside the nest from a possible intruder. However, once females had left the nest, it may have been dangerous to leave an odour trail leading back into the nest, and females resumed guarding the nest from outside. In a previous experiment with shrews, which are potential nest predators to nestling voles, we observed a similar tactic: female bank voles were outside the nest more often, but had smaller home ranges in the presence of shrews (Liesenjohann et al. 2011), probably to guard the nests from the outside. If the potential intruder is also a potential mating partner, as in our experiment, females must find a way to mate without endangering their dependent offspring, which they can achieve by mating away from the nest. In tree rats, we observed females engaging in matings with several males at a safe distance from the female’s nest (Eccard et al. 2004) and following odour trails of males (MC Breedveld et al., personal communication), again indicating that females may actively distract mating partners from the location of their offspring (Palanza et al. 1994; Jonsson et al. 2002b; Ylönen and Horne 2002) rather than defending their nest from the inside.

### Mating interest

Variables measured at the scent area (Fig. 3, Table 2) revealed a general response to the male scents independent of familiarity, which may indicate a general interest in mating and future reproduction among the females. After missing the post-partum oestrus, female bank voles seem to be able to conceive during lactation (re-analysed from Eccard and Ylönen (2003) and Eccard et al. (2017), but see Gustafsson et al. 1980, 1983) and interaction with male scents and males may trigger a behavioural oestrus, during which females were reported to actively visit males for mating (Klemme et al. 2011). In the control treatment, we found a negative correlation between litter size and the total time spent at the scent area, as well as a negative correlation with the longest visit at the scent area (Fig. 3d), probably in response to nutritional demands of the current litter. However, this relationship was not present in both male scent treatments, where females perceived a possibility to invest into a future litter (Fig. 3d), indicating that females are willing to adjust their current reproductive investment when the possibility of future reproduction arises.

### Inter-individual differences

By comparing the marginal and the conditional coefficient of determination (*R*^2^) of generalized mixed models, the importance of random factors for the predictive capacity of a model can be estimated (Nakagawa and Schielzeth 2013). In our study, the model predictions for variables related to the female’s activity (number of absences from the nest, compartments visited, and visits to the scent area) were explicitly improved (53–71% improvement of *R*^2^) by the inclusion of individual differences (i.e. female ID as random factor), while predictions of other variables (length of absence, time spent at scent area, latency to leave the nest or latency to scent) were less improved (8–40% improvement). Thus, differences among females in individual activity levels produced a lot of noise in the activity related data. If these were included in a repeated-measures design (i.e. as random factors, conditional *R*^2^), effects of treatments were more accessible to statistical modelling than without the random factor (marginal *R*^2^). Activity levels are often representative of animal personality traits (e.g. Herde and Eccard 2013); however, observed variation in activity can also be due to other life history related differences among individuals. For example, residual reproductive values (e.g. Eccard and Herde 2013) may affect the individual’s perception of value of the current litter (Williams 1966) based on individual life-history trade-offs (Pianka and Parker 1975). Since animals used in this study were wild captured, we have no information on their age or individual reproductive history, both of which may contribute to the observed differences in activity levels. The statistical inclusion of these differences using female ID as a random effect allows to investigate the general importance of treatments *within* individuals in a repeated measures design, independently from individual activity level.

### Maternal investment and behavioural trade-offs

A fundamental life history trade-off concerns the investment into current and future offspring (Stearns 1989). In our experiment, we observed behavioural trade-offs and spatial allocation potentially mediated by current reproductive investment. Females that had already invested more time and energy into their current litters, i.e. had older offspring, tended to have a higher number of absences from the nest (Table 1) and their absences were shorter for the time period until females visited the scent area for the first time (Table S2). These relationships were independent of scent treatment and may thus reflect increased nutritional demands of older litters or improved thermal regulation by older litters. With lactation being energetically very demanding (Speakman 2008), the nutritional demand of a litter may affect the behavioural trade-off between current and future reproduction. In addition to this, in the control treatment, females with larger litters spent less time at the scent wheel than females with smaller litters (Fig. 3d). However, nursing time was apparently not increased in these larger litters, since litter size did not reduce the time a female was absent from the nest. Thus, presumably females may have spent a higher proportion of their time feeding. Meanwhile, this relationship was not apparent when mating opportunities were simulated by presentation of a male’s scent, with no differences in response to male scent donors (Fig. 3d, Table 2). Possibly, females invested time into mate search when not protecting the nest. Furthermore, the time spent on searching for a mate increased when offspring were older (Fig. 3b). Rodents are short-lived and highly depredated, they maximise reproductive output and may aim to reduce the amount of time not being gravid.

## Conclusions

We investigated allocation into current and future reproduction by measuring responses of nursing bank vole females to differences in levels of infanticide risk, by mimicking the presence of the sire of her litter or of an unfamiliar male, both providing mating opportunities. Rodents are short lived, highly depredated, and have high reproductive potential. Rodent females can be concurrently pregnant and lactating, but since the experimental females were not gravid, they were thus potentially seeking a mate. Furthermore, with rodent males being potentially infanticidal to offspring that are not their own, females would have to protect offspring against unfamiliar males. We measured the females’ behaviour at the nest and found that responses to a scent-free control and to the scent of the sire of the offspring differed from responses to the scent of an unfamiliar, potentially infanticidal male. Thus, females have developed protective behaviour against potentially infanticidal, unfamiliar males. We also found that females spent more time investigating an area far from the nest when male scents were provided than under controls, irrespective of their familiarity to the male. Independent of male scent, females with older offspring visited the scent area more often indicating an interest in future reproduction in this short-lived rodent that increases with offspring age. Our results show that females appear to associate familiarity to the male with infanticide risk since they increased nest guarding in the unfamiliar treatment. Considering both observations at the scent area and nest area, our results allow the assumption that animals investing simultaneously in current and future reproduction might flexibly adjust future investment according to the levels of protection necessary for their current offspring.

## Electronic supplementary material

ESM 1 (DOCX 203 kb)
