# Supplementary material for: The scent of infanticide risk? Behavioural allocation to current and future reproduction in response to mating opportunity and familiarity with intruder
Source: Behav Ecol Sociobiol. 2018 Oct 18;72(11):175. doi: 10.1007/s00265-018-2585-4 (PMC6208815; doi:10.1007/s00265-018-2585-4)
Supplement: Supplementary file 1 — (DOCX 203 kb) [file 265_2018_2585_MOESM1_ESM.docx]

**The scent of infanticide risk? Behavioural allocation to current and future reproduction in response to mating opportunity and familiarity with intruder**

**Behavioural Ecology and Sociobiology**

**Eccard JA, Reil D, Folkertsma R, Schirmer A**

Animal Ecology, Institute of Biochemistry and Biology, University of Potsdam

Corresponding Author: eccard@uni-potsdam.de

**This article is a contribution to the Topical Collection From Sensory Perception to Behavior — Guest Editors: Theo C. M. Bakker, Horst Bleckmann, Joachim Mogdans, Vera Schlüssel**

**Supplementary Tables**

**Table S1** Descriptive statistics of response of females to male odour treatments (min: minutes, minm: minimum, max: maximum, SD: standard deviation).


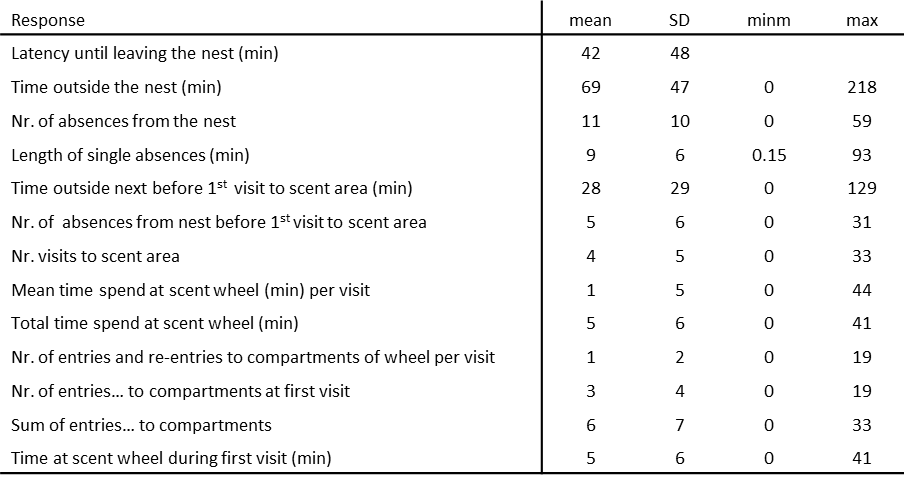


**Table S2** Models of behavioural responses at the nest of 33 vole females in 3 scent treatments (99 trials). Given are the model types (lmm: linear mixed model, data transformation (log or boxcox); glmm: generalised lmm, model family (link function)), Rm: marginal R^2^, Rc: conditional R^2^ and effect size for each fixed factor. None of the interaction of factors affected the responses, models are therefore calculated without interactions. Cum.: cumulative.

|  | |  |  |  |  |  |  |  |  |  |  |  |
| --- | --- | --- | --- | --- | --- | --- | --- | --- | --- | --- | --- | --- |
|  |  |  |  |  |  |  |  |  |  |  |  |  |
| Response Variable | Length of single absence (min) | | Cum. duration absent before 1st visit to sc. | | Cum. duration absent form nest | | Length of absence before 1^st^ visit to scent | | | Duration of longest absence | |  |
| Sample size | 1054 absences in 99 trials | | 99 trials |  | 99 trials | | 480 absences in 99 trials | | | 99 trials |  |  |
| Model | lmm(log) |  | lmm(^0.4) |  | lmm(^0.4) |  | lmm(log) | | | lmm(sqrt) | |  |
| Rm(%) | 2 |  | 4 |  | 5 |  | 4 |  |  | 3 |  |  |
| Rc(%) | 24 |  | 26 |  | 25 |  | 12 |  |  | 34 |  |  |
| Fixed factor | effect size | error | effect size | error | effect size | error | effect size | error | p | effect size | error |  |
| Sire vs control | 0.02 | 0.09 | -0.34 | 0.34 | 0.03 | 0.36 | -0.03 | 0.12 |  | 0.73 | 0.33 |  |
| Unknown vs control | -0.03 | 0.09 | -0.77 | 0.34 | 0.70 | 0.36 | 0.01 | 0.14 |  | 0.63 | 0.33 |  |
| Unknown vs sire | -0.03 | 0.09 | -0.42 | 0.34 | 0.67 | 0.36 | 0.04 | 0.14 |  | -0.10 | 0.33 |  |
| Offspring age | -0.03 | 0.05 | -0.02 | 0.05 | 0.06 | 0.06 | -0.07 | 0.03 | ** | 0.02 | 0.07 |  |
| Litter size | 0.15 | 0.09 | 0.04 | 0.13 | 0.12 | 0.14 | 0.05 | 0.06 |  | -0.05 | 0.16 |  |
|  |  |  |  |  |  |  |  |  |  |  |  |  |

**Table S3** Models of behavioural responses at the scent of 33 vole females in 3 scent treatments (99 trials). Given are the model types (lmm: linear mixed model, data transformation (log or boxcox); glmm: generalised lmm, model family (link function)), Rm: marginal R^2^, Rc: conditional R^2^ and effect size for each fixed factor. Non-significant interaction of factors were removed and models without interaction were calculated (nim: not in model).

| Response Variable | Latency |  |  | Compartments | |  | Absolute time at scent | | |
| --- | --- | --- | --- | --- | --- | --- | --- | --- | --- |
|  | to 1st scent visit | |  | at 1st scent visit | |  |  |  |  |
| Sample size | 99 trials |  |  | 99 trials |  |  |  |  |  |
| Model | lmm(^0.5) |  |  | glmm(poisson(log)) | |  | lmm(^0.4) | |  |
| Rm(%) | 5 |  |  | 51 |  |  | 15 |  |  |
| Rc(%) | 34 |  |  | 76 |  |  | 30 |  |  |
| Fixed factor | effect size | error | p | effect size | error | p | effect size | error | p |
| Sire vs control | -1.52 | 0.75 | (.) | 1.99 | 0.23 | *** | -0.97 | 0.67 | * |
| Unknown vs control | -1.53 | 0.75 | (.) | 1.61 | 0.23 | *** | -1.05 | 0.67 | * |
| Unknown vs sire | -0.01 | 0.75 |  | -0.38 | 0.23 | ** | -0.09 | 0.67 |  |
| Offspring age | -0.14 | 0.14 |  | 0.01 | 0.04 |  | 0.06 | 0.03 |  |
| Litter size | -0.12 | 0.35 |  | 0.01 | 0.10 |  | -0.27 | 0.12 |  |
| Sire*litter size (vs control) | nim |  |  | nim |  |  | 0.38 | 0.16 | * |
| Unknown*litter size (vs control) | nim |  |  | nim |  |  | 0.36 | 0.16 |  |
| Unknown*litter size(vs sire) | nim |  |  | nim |  |  | 0.03 | 0.16 |  |


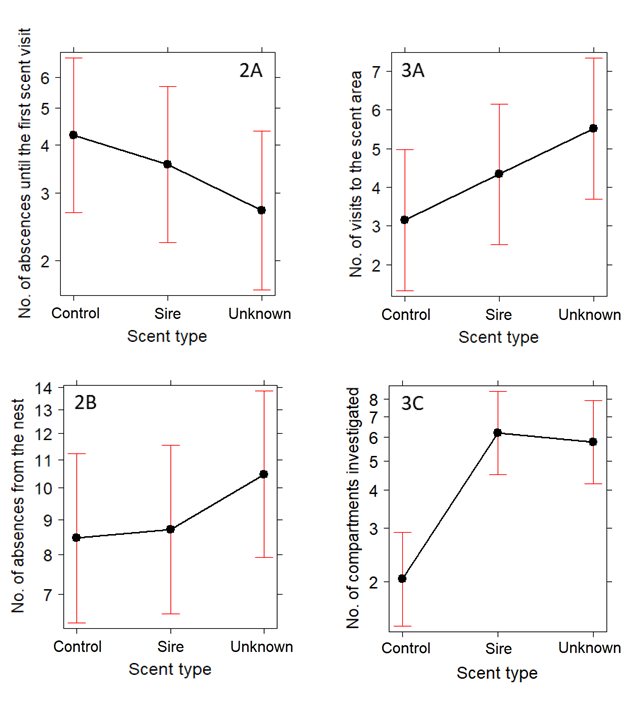


**Figure S1** Effect plots belonging to the raw data illustrations in figures 2 and 3. Represented are the effects obtained from the mixed models for the factor scent type. Black dots represent the means. Numbering refers to the respective plot in the manuscript the represented effect plot belongs to.
